# Genome-wide analysis identifies a functional association of Tet1 and Polycomb repressive complex 2 in mouse embryonic stem cells

**DOI:** 10.1186/gb-2013-14-8-r91

**Published:** 2013-08-29

**Authors:** Francesco Neri, Danny Incarnato, Anna Krepelova, Stefania Rapelli, Andrea Pagnani, Riccardo Zecchina, Caterina Parlato, Salvatore Oliviero

**Affiliations:** 1 Human Genetics Foundation (HuGeF), via Nizza 52, 10126, Torino, Italy; 2 Dipartimento di Biotecnologie Chimica e Farmacia Università degli Studi di Siena. Via Fiorentina 1, 53100 Siena, Italy; 3 Politecnico di Torino, Corso Duca degli Abruzzi 24, I-10129 Torino, Italy

**Keywords:** DNA-hydrossymethylation, Chip-Seq, PRC2, Stem Cells, Liver, Brain;Fibroblasts

## Abstract

**Background:**

Ten-Eleven Translocation (TETs)proteins mediate the oxidation of 5-methylcytosine (5mC) to 5-hydroxymethylcytosine (5hmC). Tet1 is expressed at high levels in mouse embryonic stem cells (ESCs), where it mediates the induction of 5hmC decoration on gene-regulatory elements. While the function of Tet1 is known, the mechanisms of its specificity remain unclear.

**Results:**

We perform a genome-wide comparative analysis of 5hmC in pluripotent ESCs, as well as in differentiated embryonic and adult cells. We find that 5hmC co-localization with Polycomb repressive complex 2 (PRC2) is specific to ESCs and is absent in differentiated cells. Tet1 in ESCs is distributed on bivalent genes in two independent pools: one with Sin3a centered at non-hydroxymethylated transcription start sites and another centered downstream from these sites. This latter pool of Tet1 co-localizes with 5hmC and PRC2. Through co-immunoprecipitation experiments, we show that Tet1 forms a complex with PRC2 specifically in ESCs. Genome-wide analysis of 5hmC profiles in ESCs following knockdown of the PRC2 subunit Suz12 shows a reduction of 5hmC within promoter sequences, specifically at H3K27me3-positive regions of bivalent promoters.

**Conclusions:**

In ESCs, PRC2 recruits Tet1 to chromatin at H3K27me3 positive regions of the genome, with 5hmC enriched in a broad peak centered 455 bp after the transcription start site and dependent on the PRC2 component Suz12. These results suggest that PRC2-dependent recruitment of Tet1 contributes to epigenetic plasticity throughout cell differentiation.

## Background

In eukaryotic cells, 5-methylcytosine (5mC) occurs almost exclusively within a CpG context, and is catalyzed by the family of DNA methyltransferase (DNMT) enzymes [1-3]. More recently, a number of studies have identified a mechanism of DNA demethylation involving the oxidation of 5mC to 5-hydroxymethylcytosine (5hmC), which can function as a new epigenetic marker or as an intermediate toward further oxidative states by Ten-eleven translocation (TET) proteins [4-11]. In embryonic stem cells (ESCs) and in embryonic and adult tissues, 5hmC modification occurs at high levels, whereas it is significantly reduced in cancer[12-17]. In ESCs, 5hmC is principally catalyzed by Tet1, which has high expression in these cells. Genome-wide studies in ESCs have shown 5hmC enrichment on regulatory elements, such as promoters, enhancers, and gene bodies [18-27]. Tet1 depletion in ESCs leads to both transcriptional activation, in accordance with its role in oxidation of 5mC, and transcriptional repression [6,20,25,28-32]. Genome-wide analysis have shown that Tet1 binding correlates with the transcriptional repressor Sin3a, which forms a nuclear complex with Tet1 and also with the Polycomb repressive complex 2 (PRC2) [19,23,25,32].

PRC2 is the enzymatic complex that mediates the trimethylation of lysine 27 of histone H3 (H3K27me3) on developmental genes that determine whether the chromatin remains either open or fully inaccessible. It is formed by a core complex that includes Enhancer of Zeste 1 or 2 homolog (Ezh1 or Ezh2), Suppressor of Zeste 12 homolog (Suz12), Embryonic ectoderm development (Eed), and other accessory subunits, many of which are specific to ESCs [33-41]. PRC2 is involved in a number of different biological processes, and its dysregulation is associated with carcinogenesis [33,42].

Using genome-wide analysis of 5hmC distribution in ESCs with respect to embryonic and adult tissues, we found that the overlap between the repressive modification H3K27me3 and 5hmC is ESC-specific, and we studied the molecular mechanism of the interplay between Tet1 and PRC2 in ESC.

## Results

### Genome-wide distribution of 5hmC in ESCs and differentiated cells

We mapped the genome-wide distribution of 5hmC using the glucosylation, periodate oxidation, biotinylation (GLIB) method followed by sequencing using an Illumina platform (GLIB-Seq) [18] in ESCs, primary mouse embryonic fibroblasts (MEFs), and two different tissues with distinct embryological derivation: brain, which is known to have high levels of 5hmC, and liver, which is a homogeneous tissue composed almost exclusively of hepatocytes.

**Figure 1:**
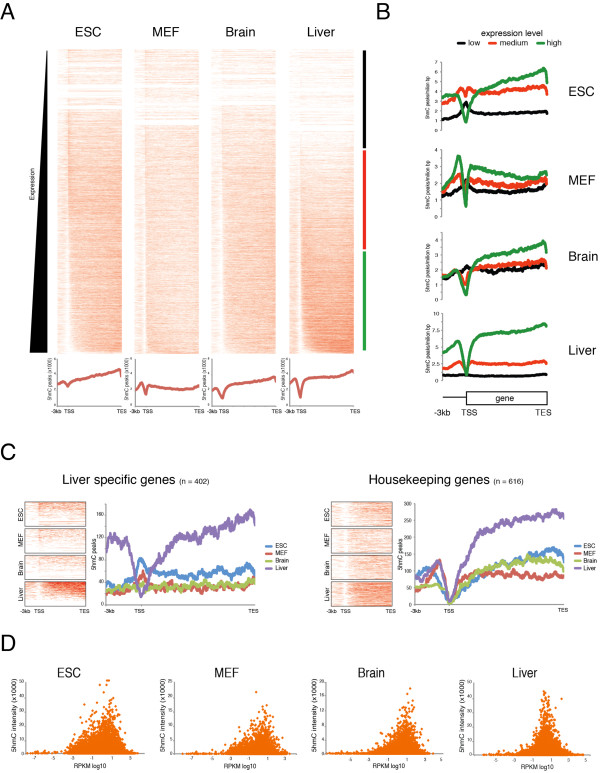
**5hmC distribution in pluripotent and differentiated cells**. **(A) **Heatmaps of 5hmC occupancy on promoters and gene bodies in embryonic stem cells (ESCs), mouse embryonic fibroblasts (MEFs), and brain and liver tissue. Genes are ordered by the mRNA levels of each cell type obtained by RNA sequencing (RNA-Seq) analysis. **(B) **The 5hmC distribution profile in promoter regions and gene bodies of genes grouped into three equal sets by their mRNA expression level. **(C) **Heatmaps and distribution profiles of 5hmC in promoters and gene bodies of genes separated into two groups according to their expression level, as expressed only in liver (liver-specific) or expressed in in ESCs, MEFs, brain, and liver (housekeeping genes). **(D) **Scatter plot of 5hmC gene body density and expression level for each gene in ESCs, MEFs, brain, and liver.

Heatmaps plotted using the gene expression levels obtained from RNA sequencing (RNA-Seq) analysis showed a similar distribution pattern in all samples, with 5hmC enrichment typically on gene bodies with respect to the transcription start site (TSS) (Figure 1A). Classification of the genes by their level of expression showed an enrichment of 5hmC on upstream promoter regions and the gene bodies of genes with higher expression. By contrast, unexpressed genes or genes expressed at low levels showed an enrichment of 5hmC at the TSS, which was more evident in undifferentiated ESCs (Figure 1B). To further investigate the relationship between hydroxymethylation and tissue specificity, we analyzed the distribution of 5hmC for genes expressed in the liver by separating them in two groups: liver-specific genes and housekeeping genes. Notably, liver-specific genes showed 5hmC enrichment only on the upstream region and along the genes in the liver itself, whereas they showed increased 5hmC at the TSSs in ESCs, MEFs, and brain tissue (Figure 1C, left panels). Conversely, the housekeeping genes in all cell types had 5hmC distributed at upstream promoter regions and in the gene bodies but not at the TSS (Figure 1C, right panels).

For each cell type, we then plotted the expression level (reads per kb of exon per million mapped reads (RPKM) log10) and the 5hmC intensity normalized for the gene length for each gene to further clarify the role of 5hmC in gene expression (see Additional file 1 Figure S1). This analysis identified 5hmC enrichment at genes with medium expression levels, with a peak around RPKM log10 value = 1 (Figure 1C), whereas genes that were not expressed (RPKM log10 value <-1) or expressed at very high levels (RPKM log10 value ≥3) showed little or no 5hmC.

The examples (see Additional file 1: Figure S1) show the 5hmC distribution and transcript levels of the *Pou5f1 *and *Albumin *genes, which are highly expressed in ESCs and liver, respectively, and *Eef1a1*, which is highly expressed in all four cell types. Taken together, these results show that 5hmC is enriched on the TSSs of genes that are not expressed or are expressed at low levels, whereas genes that are more highly expressed show an enrichment of 5hmC along the gene but not at TSSs, independent of the cell type.

### 5hmC correlates with PRC2 and H3K27me3 in ESCs, but not in differentiated cells

**Figure 2:**
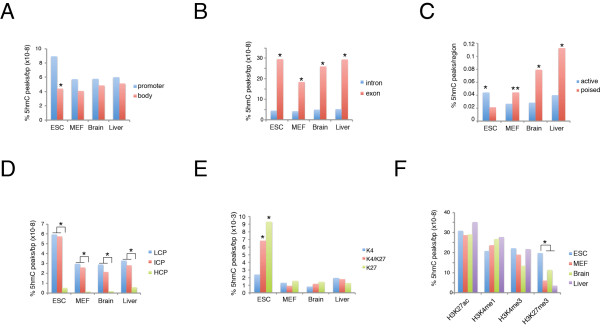
**5hmC distribution on genomic elements and histone modifications**. The percentage of 5hmC was normalized to the length (bp) of each genomic segment considered. **(A) **5hmC was enriched on promoters (± 1 kb) in embryonic stem cells (ESCs) (*P < 0.01).**(B) **5hmC was enriched on exons with respect to introns (*P < 0.01).**(C) **5hmC was significantly enriched in active enhancers in ESCs and in poised enhancers in differentiated tissues (*P < 0.01; **P < 0.05).**(D) **Promoters with low and intermediate presence of CpG islands (LCP and ICP, respectively) were preferentially marked by DNA hydroxymethylation with respect to promoters with high CpG (HCP) (*P < 0.01). **(E) **In ESCs, promoters with both H3K4me3 and H3K27me3 (K4/K27) and promoters with H3K27me3 but not H3K4me3 (K27) were significantly enriched in DNA hydroxymethylation compared with promoters with H3K4me3 but not H3K27me3 (K4) (*P < 0.01). **(F) **Analysis of correlations between 5hmC and histone modifications in all four cell types (*P < 0.01).

Recent studies in ESCs have highlighted 5hmC enrichment on specific genomic elements and in association with some histone modifications [18-20,24-26,29,32]. We extended this analysis by comparing ESCs with differentiated MEFs, and brain and liver tissues. Analysis of 5hmC density on promoters and gene bodies normalized according to base pair length showed that 5hmC was significantly enriched on promoters in ESCs but not in differentiated cells,(Figure 2A).

As previously observed, the exons in ESCs showed higher levels of DNA hydroxymethylation than did the introns, and this difference was confirmed in the other cell types we analyzed (Figure 2B). By contrast, the DNA of active enhancers was enriched by hydroxymethylation in ESCs, whereas this correlation was inverted in differentiated cells (Figure 2C). We also found significant 5hmC enrichment in promoters with low or intermediate CpG content (LCP and ICP, respectively) compared with high CpG (HCP) content in all cell types analyzed (Figure 2D).

Importantly, we found strong enrichment of 5hmC on H3K4me3K27me3 double-positive and H3K27me3-positive promoters compared with H3K4me3-positive promoters in ESCs but not in the other cell types (Figure 2E). Further comparison of our data with published histone modifications and transcription factor occupancy confirmed that, in ESCs, 5hmC co-localizes with H3K27me3, as well as with Ezh2 and Suz12 binding (Figure 2F; see Additional file 1: Figure S2)

**Figure 3:**
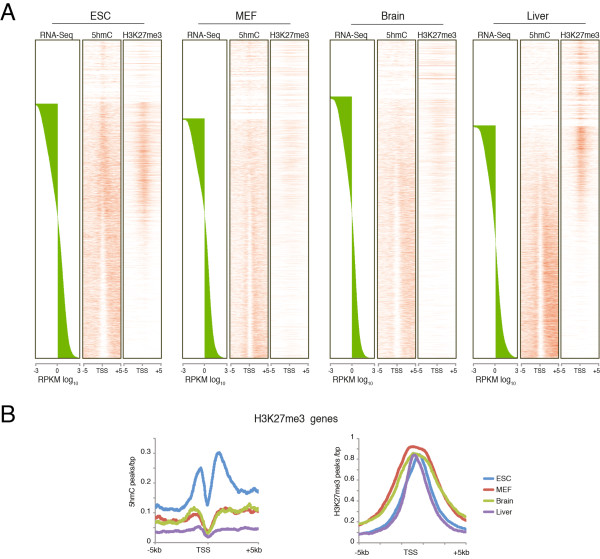
**5hmC was enriched at H3K27me3 loci in embryonic stem cells (ESCs) but not in differentiated cells**. **(A) **Heatmaps of 5hmC and H3K27me3 of genomic regions around the TSS (± 5 kb) in ESCs, mouse embryonic fibroblasts (MEFs), brain, and liver, rank-ordered by mRNA expression level of each cell type. **(B) **Profile distribution of 5hmC and H3K27me3 at the TSS (± 5kb) in H3K27me3-positive genes.

Analysis of the genome-wide distribution of 5hmC and H3K27me3 at promoters (± 5 kb) of all genes, ordered by expression level, showed that H3K27me3 is more abundant on promoters of genes with low expression in all cell types. However, in ESCs only, H3K27me3 promoters showed a significant enrichment of 5hmC (Figure 3A,B).

### Tet1 bimodal binding profile in H3K27me3 positive promoters

**Figure 4:**
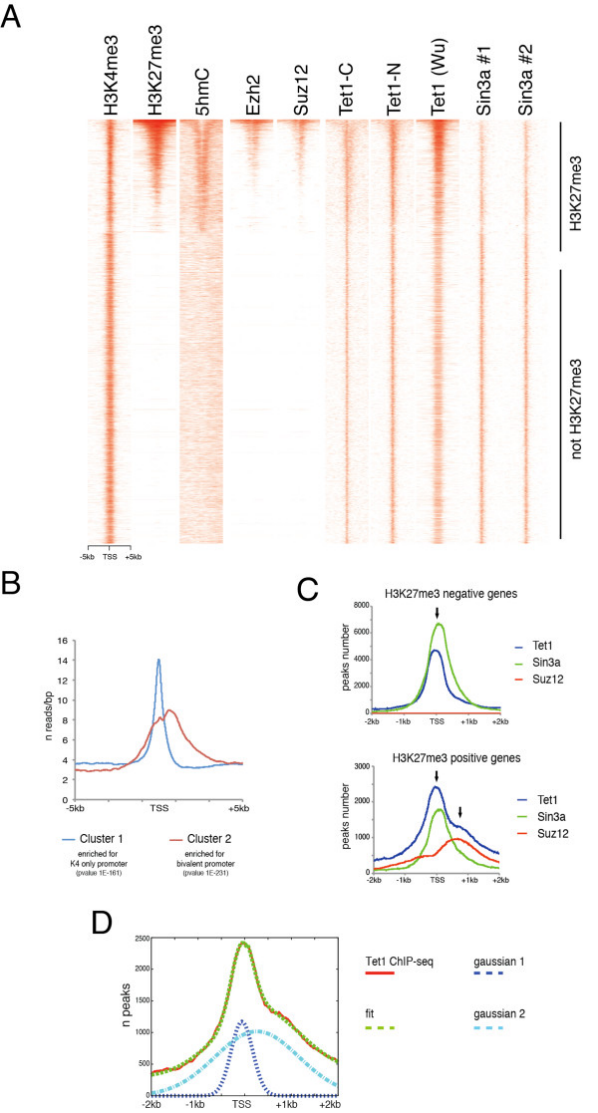
**Bimodal distribution of Tet1 on bivalent genes**. **(A) **Heatmaps of H3K4me3, H3K27me3, 5hmC, Ezh2, Suz12, Tet1-C [25], Tet1-N [25], Tet1-Wu [32], Sin3a #1 (Abcam), and Sin3a #2 (Santa Cruz Biotechnology) on the transcription start site (TSS) of genes rank-ordered by their H3K27me3 level. **(B) **Distribution frequency of Tet1 binding obtained by clustering of Tet1 binding data by k-means algorithm revealed two different Tet1 occupancy profiles on gene promoters.**(C) **Tet1, Sin3a and Suz12 occupancy around the TSS (± 2 kb) of H3K27me3-negative (upper panel) or H3K27me3-positive (lower panel) genes. **(D) **Fitting of the Tet1 binding in H3K27me3-positive genes revealed two Gaussian profiles, one centered -39 bp upstream from the TSS, and the other centered +455 bp downstream from the TSS.

The above data show a correlation between 5hmC and H3K27me3 distribution on ESC promoters. Further analysis of Tet1 distribution, using published chromatin immunoprecipitation sequencing (ChIP-Seq) datasets [25][32], on genes ordered by level of H3K27me3, showed that Tet1 binding correlates with Ezh2 and Suz12, as well as with Sin3a, a protein recently discovered in complex with Tet1 [25] (Figure 4A). Approximately 84% of Suz12 and 47% of Sin3a binding sites were also bound by Tet1, whereas the overlap between Sin3a and Suz12 was low (see Additional file 1, Figure S3).

Moreover, Tet1-Sin3a co-bound peaks showed high CpG island content, whereas the number of CpG islands was significantly decreased in Tet1-H3K27me3 co-bound regions (see Additional file 1, Figure S3).

Using unsupervised analysis of Tet1 binding, with a k-means clustering algorithm based on the Pearson correlation coefficient across profiles, we generated binding profile curves across each promoter (± 5 kb). After separating the results into two clusters (k = 2), we obtained two different Tet1 binding profiles: cluster 1, with a profile centered on the TSS and enriched at K4-only genes; and cluster 2, with a larger profile centered downstream from the TSS enriched on bivalent promoters (Figure 4B). Next, we plotted the Tet1 peaks around the TSS. On H3K27me3-negative genes, the Tet1 binding overlapped with Sin3a (Figure 4C, upper panel). Interestingly, on H3K27me3-positive genes, Tet1 displayed a bimodal profile that correlated with Sin3a on the TSS and with Suz12 downstream of the TSS (Figure 4C, lower panel). Fitting the Tet1 binding curve on the TSS (± 5 kb) using a superposition of two Gaussian distributions (six parameters fit, see Methods) showed that the first Gaussian curve was narrower and centered 39 bp upstream of the TSS, whereas the second was broader and centered 455 bp downstream of the TSS (Figure 4D). Taken together, these data suggest that Tet1 is present on bivalent genes in two distinct pools, one overlapping with Sin3a and the other with Suz12.

### PRC2 interacts with Tet1 in ESC, and its binding is required for DNA hydroxymethylation at bivalent genes

**Figure 5:**
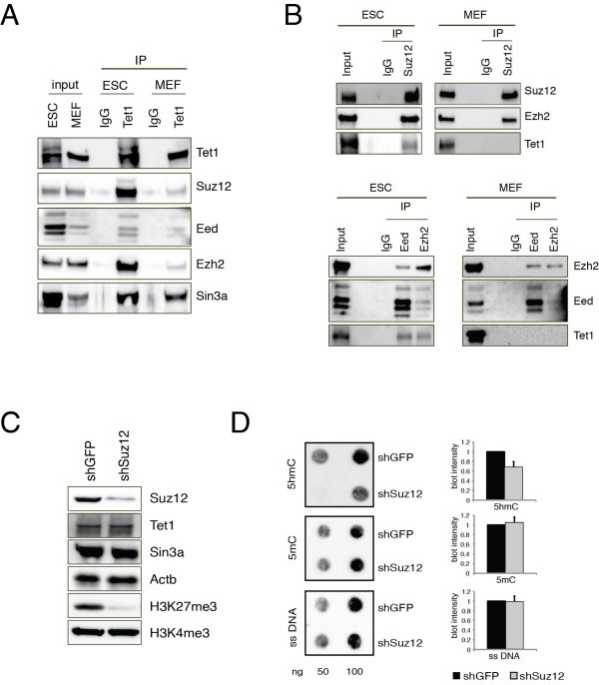
**PRC2 interacts with Tet1 and is required for correct DNA hydroxymethylation**. **(A) **Nuclear extracts of embryonic stem cells (ESCs) or mouse embryonic fibroblasts (MEFs) were immunoprecipitated with anti-IgG or anti-Tet1 antibodies. Western blotting analysis was performed using the antibodies indicated. For each input, 1% of nuclear extract was loaded. **(B) **Nuclear extracts of ESCs or MEFs were immunoprecipitated with anti-IgG or anti-Suz12 antibodies. Western blotting analysis was performed using the antibodies indicated. For each input, 2% of nuclear extract was loaded. **(C) **Western blotting analysis of extracts from the control (small hairpin green fluorescent protein; shGFP) or Suz12 knockdown (shSuz12) ESCs was performed using the antibodies indicated. **(D) **Dot-blot analysis and signal quantification of 5hmC and 5mC in DNA extracted from control or Suz12 knockdown ESCs. Single-stranded (ss)DNA was used as a loading control. The experiments were performed in triplicate.

The data above suggest interplay between Tet1 and PRC2 in ESCs. Next, we examined whether Tet1 could form a complex with PRC2 by co-immunoprecipitation of the endogenous proteins from ESCs and MEFs. Tet1 was co-immunoprecipitated with the PRC2 subunits Suz12 and Ezh2 in ESCs (Figure 5A). The reciprocal immunoprecipitation experiment with a Suz12 antibody showed association of PRC2 with Tet1 (Figure 5B). Notably, we did not observe PRC2 co-immunoprecipitation with Tet1 in MEFs (Figure 5A,B), whereas Sin3a was co-immunoprecipitated with Tet1 in both cell lines, thus confirming that the physical connection between Tet1 and PRC2 complex is limited to ESCs. To test whether the Tet1/PRC2 interaction is necessary for DNA hydroxymethylation at H3K27me3 regions, we silenced Suz12 in ESCs using two different small hairpin (sh)RNAs. Both constructs decreased Suz12 protein and H3K27me3 levels without affecting the levels of Tet1 or Sin3a (Figure 5C; see Additional file 1: Figure S3). Suz12 silencing resulted in a significant reduction of global DNA hydroxymethylation (Figure 5D; see Additional file 1: Figure S3).

**Figure 6:**
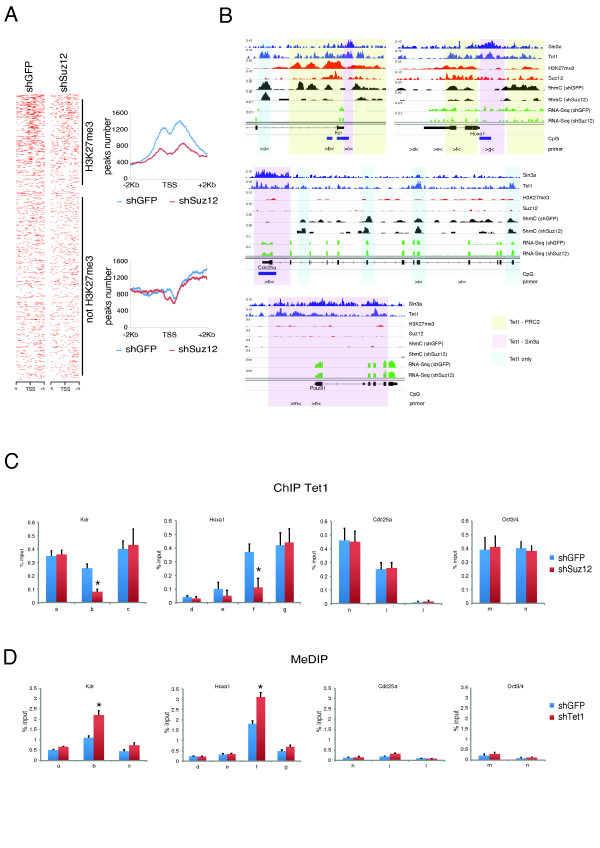
**Polycomb repressive complex 2 (PRC2) is required for 5hmC deposition and Tet1 binding in Tet1-PRC2 co-bound regions**. **(A) **Heatmaps and distribution profiles of 5hmC around the transcription start site (TSS) of genes rank-ordered by H3K27me3 levels in control or Suz12 knockdown embryonic stem cells (ESCs) See also Figure S3(C-E). **(B) **Sin3a, Tet1, H3K27me3, Suz12 occupancy and 5hmC occupancy in control and Suz12 knockdown cells on representative examples of bivalent (Kdr and Hoxa1) and K4-only (Cdc25a and Pou5f1) genes. Chromatin immunoprecipitation (ChIP) analysis of Tet1 binding of genomic regions (a to n) as indicated in panel (A), in control or Suz12 knockdown ESCs (*P < 0.01).

Genome-wide analysis of 5hmC profiles in control and Suz12 knockdown ESCs showed a reduction of 5hmC at the promoter regions of H3K27me3-positive genes within the Tet1 co-bound regions but not at H3K27me3-negative regions (Figure 6A,B; see Additional file 1, Figure S4).

ChIP analysis of Tet1 binding to the bivalent genes *Kdr *and *Hoxa1 *and the non-bivalent genes *Cdc25a *and *Pou5f1 *in wild-type and Suz12-silenced cells showed a reduction of Tet1 binding only at Tet1-PRC2 co-bound regions (Figure 6C; see Additional file 1: Figure S4). Taken together, these results indicate that PRC2 is required for recruitment of Tet1 and DNA hydroxymethylation on bivalent promoters.

## Discussion

We compared the genome-wide distribution of 5hmC in ESCs, MEF, and terminally differentiated brain and liver tissues by GLIB-Seq analysis, which identified a similar enrichment of 5hmC on promoters, enhancers, and gene bodies, with a preference for exons in all cell types. A major difference between ESCs and other cell types is the correlation between 5hmC and H3K27me3, which is unique to ESCs, whereas this correlation is not present in differentiated fibroblasts or in adult tissues. We found that this difference is due to an ESC-specific functional interplay between Tet1 and the PRC2 complex.

We found that in ESCs, but not in differentiated cells, PRC2 recruits Tet1 to the chromatin of bivalent genes to maintain their hypomethylated state. First, through in-depth analysis of previous ChIP-Seq data in ESCs, we found that Tet1 binds to chromatin with two different binding profiles: one, which is characterized by narrow peaks, is centered on the TSS of almost all genes and overlaps with Sin3a, while the other, which is characterized by broader peaks, is centered downstream of the promoters of bivalent genes and overlaps with PRC2. Second, co-immunoprecipitation showed that Tet1 interacts with PRC2 in ESCs but not in fibroblasts. Third, in ESCs, Suz12 silencing affected Tet1 binding and 5hmC modification at bivalent promoters specifically at PRC2-positive regions but not at other regions.

Thus, our results show that Tet1, besides binding at the TSS together with Sin3a, is recruited by PRC2 downstream from the TSS at bivalent genes. The difference in the peak shape of the two binding profiles is compatible with the different recruitment mechanisms. In one case, Tet1 binds directly to the DNA on the TSS via its CXXC domain, which has high affinity for clustered unmethylated CpG dinucleotides [11], whereas in the other case, Tet1 is recruited by PRC2 to the chromatin. In this latter case, the binding of Tet1 and the distribution of 5hmC occupy larger regions, owing to the typical spread of PRC2 binding on bivalent promoters. Interestingly, Tet1, together with Sin3a, localizes to unmethylated TSSs, suggesting that, at these regions, this complex binds to unmethylated CpG or catalyzes the oxidation of 5mC to completion. Conversely, Tet1 binding with PRC2 overlaps with 5hmC on bivalent genes. Thus, our results are compatible with a model by which Tet1-dependent oxidation of 5mC is finely regulated to either eliminate DNA methylation or generate 5hmC epigenetic marks in different regions. Previous findings that MeCP2, Np95, and Mbd3 recognize 5hmC [10,43-45] imply that this DNA modification can be a specific epigenetic signal.

We found that PRC2 depletion from ESCs reduced Tet1 binding on bivalent promoters. This result differs from previous studies reporting that Ezh2 knockdown did not affect Tet1 binding [32]. This discrepancy could be because depletion of Ezh2 does not impair PRC2 binding to the chromatin or its methylation activity, because in ESCs, Ezh1 can complement Ezh2 function [39]. By contrast, knockdown of Suz12 destabilizes the complex [37].

We found that Tet1/PRC2 co-immunoprecipitation is ESC-specific, as we could not co-immunoprecipitate Tet1 with PRC2 in fibroblasts, and previous experiments failed to detect PRC2 proteins interacting with Tet1 in HEK293 cells [25]. These results suggest that the functional interaction between Tet1 and PRC2 in ESCs is either indirect, possibly mediated by one of the ESC-specific cofactors, or is dependent on post-translation modifications.

We also found that the distribution of 5hmC on gene bodies is not tissue-specific, but increases with the level of gene expression in all cell types analyzed, suggesting that 5hmC acts as a positive activator by reducing the 5mC level. This regulation is confirmed by the recent finding that 5hmC was increased in gene bodies that were transcriptionally upregulated in a model of neuronal differentiation [46]. However, we found that genes expressed at very high levels showed little or no 5hmC, suggesting that DNA hydroxymethylation is not neutral but is mildly inhibitory on the transcription process. These results agree with *in vitro *experiments showing that 5hmC modifications are mildly repressive when present in the gene body [47].

We reported that PRC2 depletion reduced Tet1 binding and the presence of 5hmC at promoters of bivalent genes in ESCs. A previous report showed that Tet1 depletion results in the increased methylation and minor binding of PRC2 [32], most likely because Tet1-dependent demethylation facilitates PRC2 recruitment to the DNA, as methyl CpG counteracts the binding of PRC2. In fact, genome-wide analysis reported mutual exclusiveness of H3K27me3 with DNA methylation in CpG islands [48-51], and DNA methylation inhibits the binding of PRC2 *in vitro *[50]. Taken together, these results suggest a positive feedback loop between Tet1-dependent DNA demethylation and PRC2-dependent repression through H3K27me3 in ESCs in order to maintain developmental genes in the poised status. Thus, the presence of these two contrasting epigenetic markers on the same chromatin region is required to allow activation or repression of bivalent genes following action of developmental stimuli.

## Conclusions

Using genome-wide analysis, Suz12 silencing, and immunoprecipitation experiments we found that in ESCs, but not in other cell types, PRC2 recruits Tet1 to H3K27me3 regions. Our results identify a novel way by which Tet1 is recruited onto the chromatin, independent of its ability to bind directly to CpG or to be recruited by Sin3a, and clarify the mechanism that links H3K27me3 modifications on the nucleosome and 5hmC on the DNA in ESCs. These findings highlight the mechanistic link between PRC2 and Tet1 and contribute to our understanding of how these two epigenetic modifiers regulate the chromatin state of bivalent genes to maintain these bivalent genes in a poised state, ensuring their dynamic regulation during differentiation.

## Materials and methods

### Cell culture

E14 mouse ES cells were cultured in high-glucose DMEM (Invitrogen Corp., Carlsbad, CA, USA) supplemented with 15% FBS (Millipore Corp., Billerica, MA, USA), 0.1 mmol/l nonessential amino acids (Invitrogen), 1 mmol/l sodium pyruvate (Invitrogen), 0.1 mmol/l 2-mercaptoethanol, 1500 U/ml Leukemia Inhibitory Factor (LIF; Millipore), 25 U/ml penicillin, and 25 µg/ml streptomycin. MEFs were derived from 13.5 day pregnant female mice and cultured in high-glucose DMEM supplemented with 10% FCS. Mouse tissues were extracted from 8-week-old mice. For silencing of Suz12, ESCs were transfected as previously described [52] with the TRCN0000123889 and TRCN0000123891 vectors (Open Biosystems/Thermo Scientific Inc., Ptitsburgh PA, USA) and then maintained for selection for 3 days with 1 µg/ml puromycin.

### DNA extraction and GLIB-Seq

Genomic DNA was extracted using a DNeasy Blood and Tissue kit (Qiagen Inc., Valencia, CA, USA).

GLIB was precipitated using a Hydroxymethyl Collector (Active Motif, Carlsbad, CA, USA). Libraries were generated with a ChIP-Seq Sample Prep Kit (Illumina Inc., San Diego, CA, USA) and sequenced on an HiScanSQ Platform (Illumina) .

### Bioinformatic analysis

Sequencing data were mapped to the mouse genome (mm9 assembly) using Bowtie (version 0.12.7), reporting only unique hits with up to two mismatches. Redundancies were collapsed, and peak calling was performed using MACS (version 1.4.1). For comparative analysis, we downloaded GEO Datasets data for the ESC histone modifications GSE12241 [53,54] and GSE11172 [55], and the transcription factors GSE11431 [56], GSE24843 [25], and GSE26833 [19,32]. For histone modifications and mRNA expression, ChIP-Seq and RNA-Seq data on ESCs, MEFs, and brain or liver tissues were downloaded from the ENCODE project database [57], corresponding to the following GEO Datasets: GSE36025, GSE36026, and GSE31039. Datasets for ChIP-Seq of H3K27me3 on MEFs was downloaded from GSE12241.

LCP-ICP-HCP promoters were defined as described by Weber *et al*. [58], H3K4me3-only and bivalent promoters were defined as described by Ku *et al*. [59], and active or poised enhancers were defined as previously described [60,61]. To obtain three groups of equal size, genes with RPKM less than 0.1, 0.1 to 5, and less 5 were categorized as having no, low, and medium/high expression, respectively. Liver-specific genes were defined as having RPKM less than 10 in liver and less than 1 in other cells. Housekeeping genes were defined as RPKM greater than 10 in all cell types.

Data mapped on the mouse mm8 assembly was transposed to the mm9 assembly using the liftOver tool. Heatmaps and comparative analyses were performed using custom Perl scripts. Clustering of Tet1 binding profiles was performed using the MATLAB implementation of the k-means algorithm using the Pearson correlation coefficient as the 'distance' between profiles. Using 100 realizations of the clustering (using different random seeds), we foudn that the composition of clusters was remarkably stable. The fitting procedure of the average Tet1 binding profile was performed using the gnuplot least squares fitting algorithm. The test function was a superposition of two (non-normalized) Gaussian distributions (six parameters fit: two means, two variances, one relative weight, one overall amplitude factor).

### Nuclear protein extractions

Cells were harvested in 1× PBS and resuspended in isotonic buffer (20 mmol/l HEPES pH 7.5, 100 mmol/l NaCl, 250 mmol/l sucrose, 5 mmol/l MgCl_2_, 5 µmol/l ZnCl_2_), then cells were resuspended in isotonic buffer supplemented with 1% NP-40 to isolate nuclei. The isolated nuclei were resuspended in digestion buffer (50 mmol/l Tris-HCl pH 8.0, 100 mmol/l NaCl, 250 mmol/l Sucrose, 0.5 mmol/l MgCl2, 5 mmol/l CaCl_2_, 5 µmol/l ZnCl_2_), and treated with microccocal nuclease at 30°C for 10 min.

### Immunoprecipitations

Nuclear proteins were incubated with 3 µg of specific antibody overnight at 4°C. Immunocomplexes were incubated with Protein G-conjugated magnetic beads (Dynal; Invitrogen) for 2 h at 4°C. Samples were washed four times with digestion buffer supplemented with 0.1% NP-40 at room temperature. Proteins were eluted by incubating with 0.4 M NaCl TE buffer for 30 min, and analyzed by western blotting as previously described [62].

### Chromatin immunoprecipitation and dot-blot analysis

Each ChIP experiment was performed at least three independent times, as previously described [52]. Oligonucleotide sequences are shown in the supplementary data. See Additional file 1. Genomic DNA for the dot-blot analysis was sonicated for 15 cycles and denatured with 0.4 M NaOH, then incubated for 10 minutes at 95°C before being spotted onto Hybond™-N^+ ^membranes (GE Healthcare, Little Chalfont, Buckinghamshire, UK).

### Antibodies

The antibodies used were anti-Tet1, anti-H3K27me3, anti-H3K4me3 (all Millipore), anti-ssDNA (Abcam, Cambridge, MA, USA), anti-Suz12and anti-Ezh2 (bothCell Signaling Technology, Danvers, MA, USA), anti-actin (Sigma-Aldrich, St Louis, MO, USA), anti-Sin3a (SantaCruz Biotechnology Inc., Santa Cruz, CA, USA), and anti-5mC and anti-5hmC (both Active Motif).

### Data access

The Gene Expression Omnibus accession number for 5hmC profiling of ESC, MEF, brain and liver reported in this paper is GSE44566.

## Abbreviations

5mC: 5-methylcytosine; 5hmC: 5-hydroxymethylcytosine; ChIP: chromatin immunoprecipitation; Eed: Embryonic ectoderm development; ESC. Embryonic stem cell; Ezh1: Enhancer of Zeste homolog 1; GLIB: Glucosylation: periodate oxidation: biotinylation; HCP: High CpG; ICP: Intemediate CpG; LCP: Low CpG; MEF: Mouse embryonic fibroblast; PRC2. Polycomb repressive complex 2; RPKM: Reads per kb of exon per million mapped reads; Suz12: Suppressor of Zeste 12 homolog; TET: Ten-eleven translocation; TSS: Transcription start site

## Authors' contributions

FN and SO designed the experiments and co-wrote the paper. FN and DI performed the ChIP-Seq experiments and analyzed the data. AP and RZ contributed to the bioinformatic analysis. AK performed the tissue preparations and the biochemical experiments. CP and SR contributed to cell culture and DNA cloning. All authors read and approved the final manuscript.

## Supplementary Material

Additional file 1 **PDF document containing four supplemental figures (Figures S1 to S4), and one supplemental table (Table S1)**. Click here for file
