# Supplementary material for: Genome-wide analysis identifies a functional association of Tet1 and Polycomb repressive complex 2 in mouse embryonic stem cells
Source: Genome Biol. 2013 Aug 29;14(8):R91. doi: 10.1186/gb-2013-14-8-r91 (PMC4053938; doi:10.1186/gb-2013-14-8-r91)
Supplement: Additional file 1 — PDF document containing four supplemental figures (Figures S1 to S4), and one supplemental table (Table S1). [file gb-2013-14-8-r91-S1.PDF]

Additional File 1

Supplemental Information for Neri et al. “Genome-wide analysis identifies a functional association of Tet1 and Polycomb PRC2 in mouse embryonic stem cells”.

This file contains 4 supplemental figures and 1 supplemental table.

A

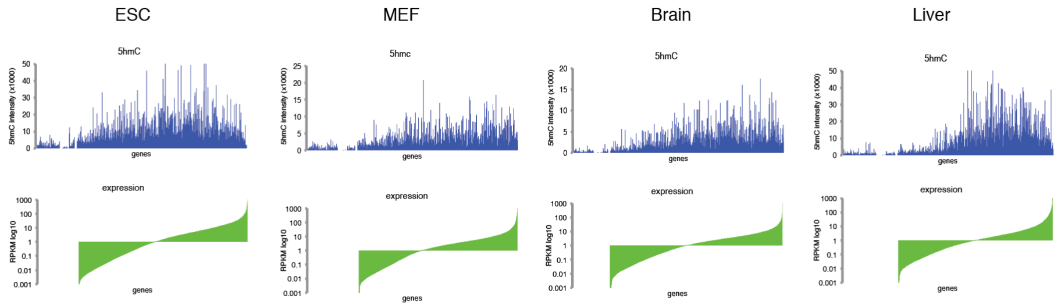

B

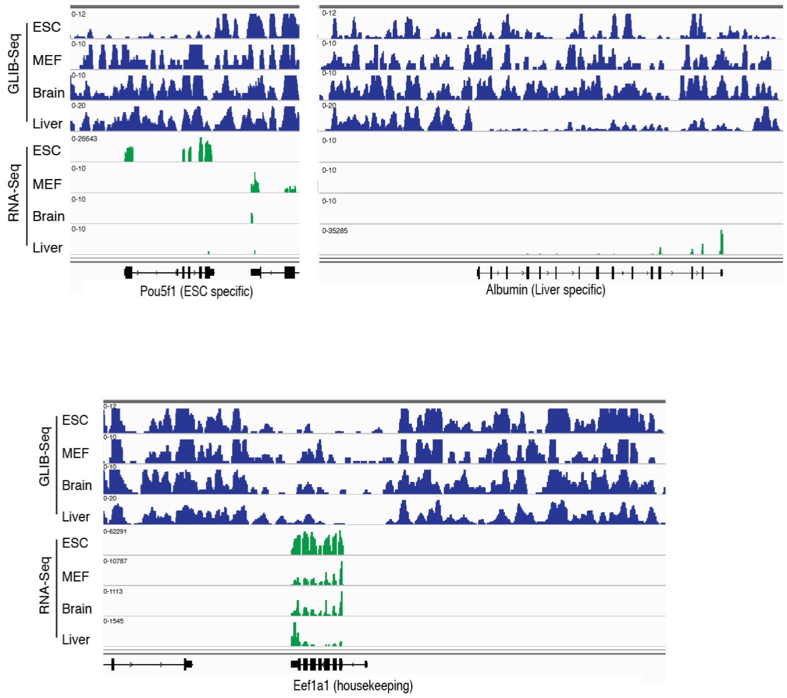

Figure S1. 5hmC is Enriched at Genes Expressed at Medium Level
(A) 5hmC intensity quantification and mRNA expression level for each gene in ESCs, MEF, Brain and Liver. 5hmC quantification was made by counting the number of reads obtained from GLIB-Seq and normalized for number of base pairs for each gene. The graph is rank-ordered from less expressed gene to most expressed gene.
(B) Genomic view of 5hmC occupancy and RNA-Seq profiles of some representative, highly expressed genes: Pou5f1 (ESC specific), Albumin (Liver specific) and Eef1 (common to all cell types).

A

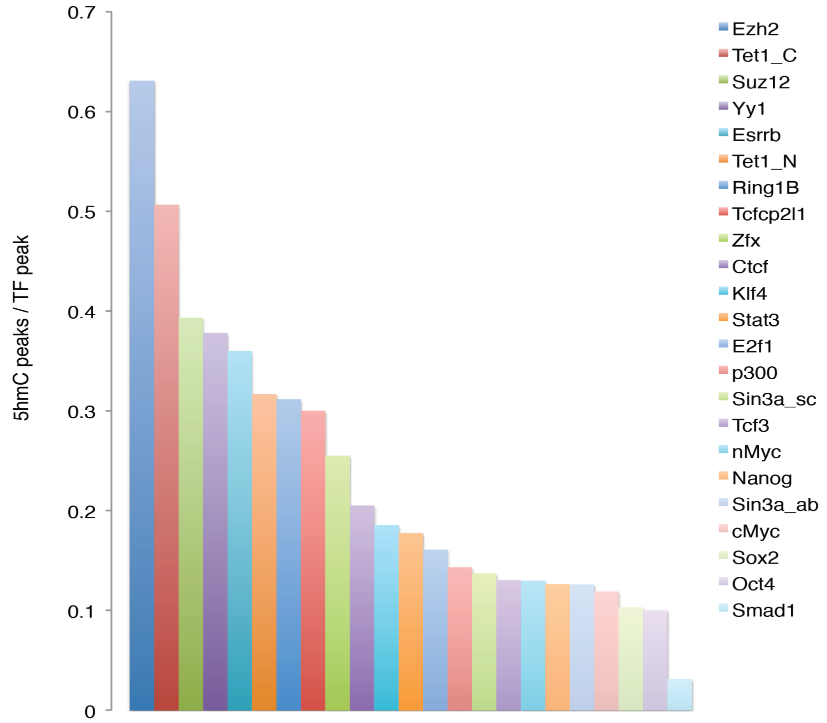

B

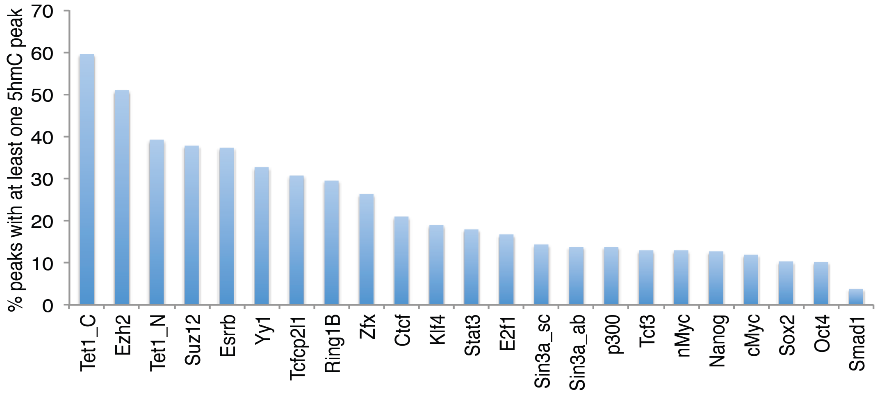

Figure S2. Correlation between 5hmC occupancy and Transcriptional Factors (TF) binding in ESCs.
(A) The Y-axis represents the average number of hydroxymethylated regions overlapping the region of binding of each of the indicated transcription factors in ESCs.
(B) In this graph, the percentage of the indicated transcription factors bound regions having at least one hydroxymethylated region is shown.

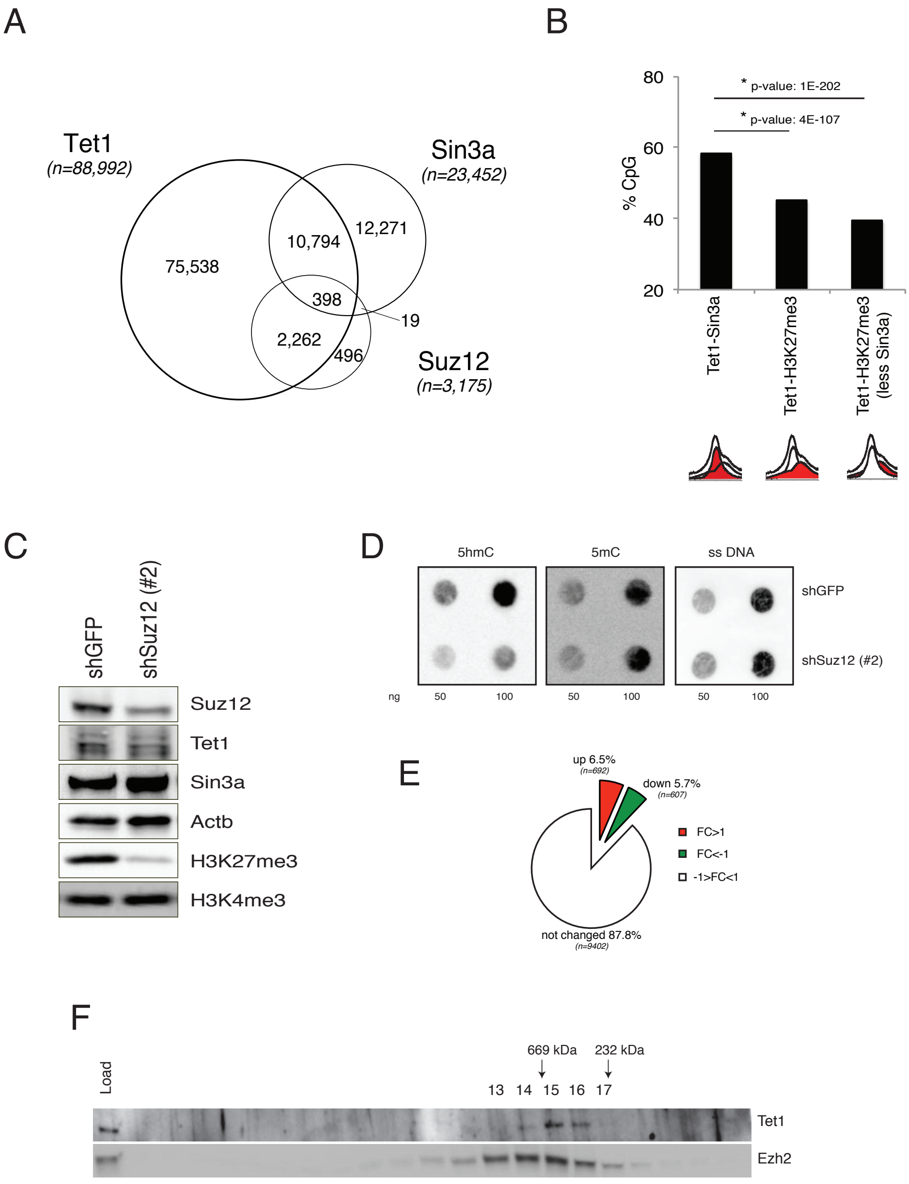

Figure S3. PRC2 Interacts with Tet1 and Is Required for a Correct DNA hydroxymethylation on Bivalent Genes

(A) Venn diagram of Tet1, Sin3a and Suz12 binding sites in ESCs. ChIP-seq raw data were re-mapped with the same software and the bound regions were re-called using MACS with the default parameters and p-value equal to p1E-8.

(B) Percentage of CpG islands in regions co-bound by Tet1 and Sin3a or Tet1 and H3K27me3 or Tet1 and H3K27me3, but not Sin3a. CpG islands coordinates for mm9 genome assembly were downloaded from UCSC database.

(C) Western blot analysis showing the levels of Suz12, Tet1, Sin3a and H3K27me3 in control or knockdown Suz12 (using shRNA #2) cells. Actin and H3K4me3 were used as a loading control.

(D) Dot-Blot analysis of 5hmC and 5mC of control or Suz12 knockdown ESCs using a different shRNA (shRNA #2). ssDNA was used as a loading control.

(E) Diagram showing the percentage of genes unchanged, up- or downregulated.

(F) Gel filtration of ESC nuclear extract performed using Superose 6 10/300GL column.

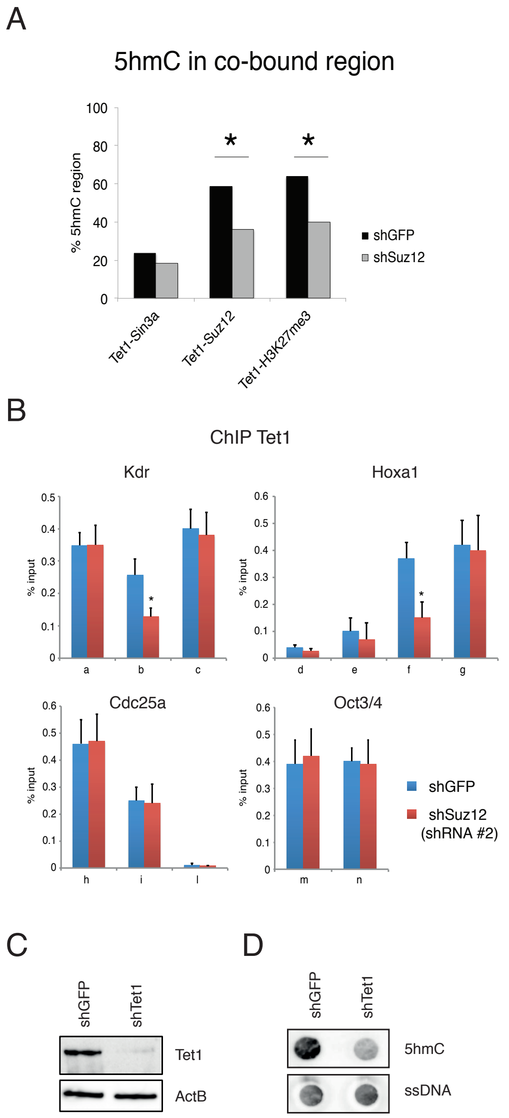

Figure S4. PRC2 Is Required for Tet1 Binding on Bivalent Genes

(A) Percentage of 5hmC positive regions in Tet1 co-bound regions with Sin3a, Suz12 and H3K27me3 in control and Suz12 knockdown ESCs. (*p-value <0.001).

(B) ChIP analysis of Tet1 binding of indicated region (a-n) in control or Suz12 knockdown ESCs using a different shRNA. Position of primer (a-n) is indicated in Figure 6B. The regions that show a significant decrease of Tet1 binding ("b" and "f") are downstream of TSS of the relative genes. In genome-wide analysis, these regions do not show binding of Sin3a, but show a high level of DNA hydroxymethylation. * p-value < 0.01

(C) Western blot analysis showing the level of Tet1 in control or knockdown Tet1 cells. Actin was used as a loading control.

(D) Dot-Blot analysis of 5hmC of control or Tet1 knockdown ESCs. ssDNA was used as a loading control.

Table S1: Primer list

Gene	Primer	Strand	Sequence
Kdr	a	fw	CAATGCACACTCCAAGTTTT
Kdr	b	fw	TTGAAGCAGAGGGCATTTTA
Kdr	c	fw	AACACAGCTTACTCTCTTGG
Hoxa1	d	fw	AGAAGCAGACAGCAAAGTTA
Hoxa1	e	fw	TTAAGACCCGTAAACTCTGC
Hoxa1	f	fw	GCAAACTCACACACATGAAA
Hoxa1	g	fw	GGAATCCAACAGACACCAC
Cdc25a	h	fw	GAAGTTTGCTTCCTGATTGG
Cdc25a	i	fw	TTAATCCAAGGTTGGACCTG
Cdc25a	l	fw	TTAGGATCATGTGAAGCTGG
Oct3/4	m	fw	TCCTCCTAATCCCGTCTCCT
Oct3/4	n	fw	CTGTAAGGACAGGCCGAGAG
Kdr	a	rev	GTTCATCGGGTTTCAATGTC
Kdr	b	rev	ACTTTCCTCCCTCAACTTTC
Kdr	c	rev	TCCAGATTTGCTCTCAGATG
Hoxa1	d	rev	TTTCTGAGGCAAGTCCATAC
Hoxa1	e	rev	TGGATGGCACAATGAATGTA
Hoxa1	f	rev	CTCCCCACAACCTATGATTC
Hoxa1	g	rev	TAAGACCCTTTGGATGAGGA
Cdc25a	h	rev	AAATCCAAACAAACTCCGTG
Cdc25a	i	rev	TGGATCAAGGGAGAAGGTAA
Cdc25a	l	rev	CTGTGAACCTCTTTCTTCCA
Oct3/4	m	rev	TCCCCTCACACAAGACTTCC
Oct3/4	n	rev	CAGGAGGCCTTCATTTTCAA

List of primers used for Tet1 ChIP analysis
